# Glycaemic variability, assessed with continuous glucose monitors, is associated with diet, lifestyle and health in people without diabetes

**DOI:** 10.21203/rs.3.rs-3469475/v1

**Published:** 2023-10-30

**Authors:** Kate M. Bermingham, Harry A. Smith, Javier T. Gonzalez, Emma L Duncan, Ana M. Valdes, Paul W. Franks, Linda Delahanty, Hassan S. Dashti, Richard Davies, George Hadjigeorgiou, Jonathan Wolf, Andrew T. Chan, Tim D. Spector, Sarah E. Berry

**Affiliations:** 1 Department of Nutritional Sciences, King’s College London, London, UK.; 2 Zoe Ltd, London, UK.; 3 Centre for Nutrition, Exercise, and Metabolism, Department for Health, University of Bath, UK.; 4 Dept of Diabetes and Endocrinology, Guy’s and St Thomas’ NHS Foundation Trust, London, UK; 5 School of Medicine, University of Nottingham, Nottingham, UK.; 6 Nottingham NIHR Biomedical Research Centre, Nottingham, UK.; 7 Department of Clinical Sciences, Lund University.; 8 Department of Anesthesia, Critical Care and Pain Medicine, Massachusetts General Hospital and Harvard Medical School, Boston, MA, USA; 9 Diabetes Center, Department of Medicine, Massachusetts General Hospital, Boston, MA, USA.; 10 Department of Medicine, Harvard Medical School, Boston, MA, USA.; 11 Clinical and Translational Epidemiology Unit, Massachusetts General Hospital, Boston, MA, USA.; 12 Department of Twin Research and Genetic Epidemiology, King’s College London, London, UK.

## Abstract

**Background:**

Continuous glucose monitors (CGMs) provide high-frequency information regarding daily glucose variation and are recognised as effective for improving glycaemic control in individuals living with diabetes. Despite increased use in individuals with non-diabetic blood glucose concentrations (euglycemia), their utility as a health tool in this population remains unclear.

**Objectives:**

To characterise variation in time in range (TIR) and glycaemic variability in large populations without diabetes or impaired glucose tolerance; describe associations between CGM-derived glycaemic metrics and metabolic and cardiometabolic health traits; identify key diet and lifestyle factors associated with TIR and glycaemic variability.

**Design:**

Glycaemic variability (coefficient of variation) and time spent in both the ADA secondary target range (TIR_ADA_; 3.9–7.8 mmol/L) and a more stringent range (TIR_3.9–5.6_; 3.9–5.6 mmol/L) were calculated during free-living in PREDICT 1, PREDICT 2, and PREDICT 3 euglycaemic community-based volunteer cohorts. Associations between CGM derived glycaemic metrics, markers of cardiometabolic health, diet (food frequency questionnaire and logged diet records), diet-habits, and lifestyle were explored.

**Results:**

Data from N=4135 participants (Mean SD; Age: 47 12 y; Sex: 83% Female, BMI: 27 6 kg/m^2^). Median glycaemic variability was 14.8% (IQR 12.6–17.6%), median TIR_ADA_ was 95.8% (IQR 89.6–98.6%) and TIR_3.9–5.6_ was 75.0% (IQR 64.6–82.8%). Greater TIR_3.9–5.6_ was associated with lower HbA1c, ASCVD 10y risk and HOMA-IR (all p < 0.05). Lower glycaemic variability was associated with lower % energy derived from carbohydrate (r_s_: 0.17, p < 0.01), ultra-processed foods (NOVA 4, % EI; r_s_: 0.12, p = 0.01) and a longer overnight fasting duration (r_s_: −0.10, p = 0.01).

**Conclusions:**

A stringent TIR target provides sensitivity to detect changes in HOMA-IR, ASCVD 10 y risk and HbA1c that were not detected using ADA secondary targets. Associations among TIR, glycaemic variability, dietary intake (e.g. carbohydrate and protein) and habits (e.g. nocturnal fasting duration) highlight potential strategic targets to improve glycaemic metrics derived from continuous glucose monitors.

## Introduction

Continuous glucose monitors (CGMs) provide real time data of fluctuations in interstitial fluid glucose throughout the day in free-living conditions ^1,2^. Compared to traditional blood glucose monitoring, such as capillary fingertip or venous glucose measurements, CGMs provide high-frequency assessments of glucose variation, including magnitude, duration, and frequency of within and between-day glucose variability^1^. In people living with diabetes, CGMs can help improve glycaemic control^3^. Greater exposure to elevated postprandial glucose, indicated by time spent above range (>10.0 mmol/L), precedes changes in fasting glucose and HbA1c that indicate dysregulated glucose metabolism ^4^. Therefore, CGM-derived metrics are more discriminatory of deterioration in blood glucose control than HbA1c and are also linked to arterial wall thickening ^2–7^. Consequently, CGMs have become common in diabetes management, providing an effective means for detecting impaired glucose regulation and helping to set realistic treatment goals ^1,2^. However, the health utility of CGMs in individuals with normal glycaemia remains unclear.

Despite the increase in use of CGMs in individuals with euglycaemia there is currently very limited published data on glycaemic variability and health outcomes in this population. However, postprandial glucose control is relevant, even in people with euglycaemia, as a glucose concentration at 2 hours post oral glucose tolerance test (OGTT) that remains above fasting levels is associated with increased risk of CVD events ^8^. Moreover, standard tests to assess glucose control such as fasting glucose and OGTT provide little insight into daily glucose exposure, and HbA1c lacks insight into the dynamics of glucose fluctuations over time ^4,9^. In contrast, the multiple features that CGM’s measure capture the net effects of underlying physiology and acute behaviours.

Common CGM-derived metrics include time in range (TIR) and glycaemic variability. Individuals without IGT or type 2 diabetes spend 93–99% of their time within the established ADA TIR target ^10–19^. However, a random plasma glucose (RPG) concentration of 5.6 mmol/L shows high sensitivity and specificity for identifying people in need of diabetes screening relative to the American Diabetes Association (ADA) and U.S. Preventive Services Task Force (USPSTF) screening strategies ^20–22^. In contrast, individuals with euglycaemia spend ~20% of time above 5.6 mmol/L, and ~80% of time between 3.3 to 5.6 mmol/L ^10^. Whilst it is possible that the above evidence may reflect glucose concentrations as an indirect marker rather than direct cause, a direct causal role of glycaemic variability as a factor in cardiometabolic health within the euglycaemic range has also not been excluded. Consequently, there is a need to examine possible relationships between glycaemic metrics and health markers within a lower specified range.

In addition to elucidating the role of glycemic variation in cardiometabolic health, CGMs may contribute to understanding the influence of diet and lifestyle on glycaemic control in individuals without diabetes. Associations between measures of glycaemic variability, biomarkers of cardiometabolic health, dietary composition, and sleep quality have been recently reported ^19^, highlighting the potential of CGMs to identify perturbations that may influence longer term health in individuals without diabetes. Additionally, glucose homeostasis and cardiometabolic health are influenced by other dietary and lifestyle factors such as food structure, meal timing, eating patterns, and physical activity ^23–27^. Additional insight can be ascertained through assessment of the relationship between a standardised OGTT to assess the relationship to glucose tolerance. Remaining questions around CGM use in populations without diabetes therefore include the exploration of any link between glycaemic metrics and cardiovascular disease intermediary risk measures, as well as dietary and lifestyle factors.

The PREDICT studies, which enrolled a large number of participants who provided CGM data, offer such an opportunity to explore the relationship between glycaemic variability and cardiometabolic health in people without diabetes. Accordingly, the aim of this research was to 1) examine variation in TIR and glycaemic variability in populations without diabetes or impaired glucose tolerance; 2) understand associations between CGM-derived glycaemic metrics with metabolic and cardiometabolic health measures; and 3) identify diet and lifestyle factors associated with TIR and glycaemic variability. We hypothesised that glycaemic variability and time in range would be associated with cardiometabolic health measures, diet, and lifestyle in the euglycaemic range.

## Materials and methods

The ZOE PREDICT programme of personalised nutrition research consists of several diet intervention studies examining relationships between diet and cardiometabolic health. PREDICT 1 (n=1,002) (NCT03479866) was the first intervention study in the United Kingdom (UK) aiming to derive algorithms that predict an individual’s postprandial metabolic responses to specific foods ^28,29^. The primary outcomes were variations in serum concentrations of triglyceride, glucose and insulin in response to sequential mixed-nutrient dietary challenges during a clinical visit. Secondary outcomes included CGM, lipemic C-peptide responses assessed by dried blood spot (DBS) cards collected at home across three postprandial time points over the subsequent 13-day home-testing phase. Ethical approval was granted by the London-Hampstead Research Ethics Committee (approval no. 18/LO/0663) and Integrated Research Application System (no. 236407). The study was run in accordance with the Declaration of Helsinki and good clinical practice; and all participants provided informed written consent upon enrolment. Exclusion criteria included ongoing inflammatory disease; cancer in the last three years (excluding skin cancer); long-term gastrointestinal disorders including inflammatory bowel disease or Coeliac disease, but not including irritable bowel syndrome; taking immunosuppressants or antibiotics as daily medication within the last three months; capillary glucose level of >12 mmol/L (or 216 mg/dL), or type 1 diabetes mellitus, or taking medication for type 2 diabetes mellitus; currently experiencing acute clinically diagnosed depression; myocardial infarction or stroke in the last 6 months; pregnant; and vegan or experiencing an eating disorder or unwilling to consume foods that are part of the study. A group of participants who underwent additional cardiometabolic phenotyping (*n*=49) were also included in this cohort. The PREDICT 1 cohort (*n*=1002) and the additional cardiometabolic group (*n*=49) were combined and are referred to collectively as the PREDICT 1 cohort from here out (n=1,051).

### Baseline characteristics and clinical chemistry markers.

Demographic and clinical measures selected for this research included: sex, age, height, weight, fasting plasma glucose, HbA1c, total cholesterol, high-density lipoprotein cholesterol (HDL-C) and triglyceride (TG) concentrations. Descriptive information including sex and age were self-reported. For body composition measures, height and weight were measured at a clinical visit (day 0). Considering clinical biochemistry: fasting venous blood samples were collected at the clinical visit; plasma glucose and serum total cholesterol, HDL-C and TG were measured by Affinity 1.0 laboratory, and whole blood HbA1c by Viapath laboratory. The 10-year atherosclerotic cardiovascular disease (ASCVD) risk was calculated per the 2019 ACC/AHA guidelines (19). Additional data were collected over the subsequent 13-d period at home: postprandial responses to eight standardised meals (seven in duplicate) of differing macronutrient (fat, carbohydrate, protein, and fibre) content, including an oral glucose tolerance test (OGTT), were measured using continuous glucose monitors (CGMs) and dried-blood-spot (DBS) analysis.

### CGM devices.

Participants were fitted with wearable CGM devices (Abbott Freestyle Libre Pro (FSL; Abbott, Abbott Park, IL, US)) at the clinic visit (day 0). CGMs were fitted on the upper arms and covered with Opsite Flexifix adhesive film (Smith & Nephew Medical Ltd, Hull, England) for improved durability. Calibration was not required. Subcutaneous tissue interstitial fluid glucose levels were recorded every 15 minutes, and monitors captured interstitial fluid glucose concentrations between 2.2–22.2 mmol/L. PREDICT 1 participants were fully blinded to their CGM results. CGMs were worn by participants for 14 days, of which 2–4 days were free-living days (study days 10–14) meaning no standardised meals were consumed. CGM data were collected in coordinated universal time (UTC) and later adjusted to participant-specific time zones. Participants with inadequate CGM data and excluded from analysis included those with 1) <2 free-living days, 2) >1 time zone during the free-living period, 3) CGM malfunction as classified by >25% of readings at monitor baseline per day or >10% missing reads per day, or 4) glucose intolerance, defined by a fasting plasma glucose ≥6.1 mmol/L (110 mg/dL) or oral glucose tolerance test >7.6 mmol/L or 5) HbA1c levels >6.5% (48 mmol/mol). All interstitial fluid glucose levels are reported as means with SD. Glucose variability was measured by the coefficient of variation (%), calculated as the average of daily CVs (SD divided by the mean). TIR was calculated based on two cut-off values: 1) the American Diabetes Association (ADA) secondary cut-offs (TIR_ADA_; 3.9–7.8 mmol/L) ^2^, and 2) an exploratory range TIR cut-off (TIR_3.9–5.6_; 3.9–5.6 mmol/L), defined by us based on previously published data ^30^, to visualise glycaemic variability in participants without type 1 diabetes, type 2 diabetes mellitus or IGT.

### Dietary data.

Pre-enrollment, participants completed the European Prospective Investigation into Cancer and Nutrition (EPIC) food frequency questionnaire (FFQ) to capture their past-year intake of foods ^31^. Habitual food intake, energy expenditure and nutrients were ascertained from the FFQ as previously described ^28^. Free-living logged diet data was also collected from which food intake, energy expenditure and nutrients were collected on free-living days with concurrent CGM data. Nutrient values were obtained for 11,333 foods of 42,211 foods from the McCance and Widdowson Food and Nutrient database ^32^. For branded foods, nutrient information was collated from common supermarket websites. A day was excluded per participant when daily calorie intake fell outside gender specific cut-offs deemed implausible (females; 500–5000 kcal and males; 500–8000 kcal). Plant based diet index scores were calculated as a measure of diet quality ^33^. Percentage contributions to total daily energy were calculated for protein, carbohydrate, and fat as well as unprocessed foods and ultra-processed foods using the NOVA classification system ^34^.

Several additional diet habit variables were calculated using the free-living logged diet data. An eating occasion (EO) was defined as any occasion where a food or beverage was consumed that contained ≥50 kcal and was separated in time from the preceding and succeeding EO by 30 minutes ^35,36^. A main meal was defined as ≥400 kcal for females and ≥500 kcal for males ^37^. Snacks were defined as eating occasions that were not main meals (females; 50–399 kcal, males 50–499 kcal). First and last eating occasion times were the time of day when the first and last eating occasion occurred (≥50kal) ^38^. Fasting window was calculated as the difference between the last and first eating occasion. Eating midpoint was the midpoint in time between the first and last eating occasion. All dietary habit variables were calculated for each free-living day and mean free-living values were calculated.

### Activity and sleep.

Activity energy expenditure (AEE), sleep duration (i.e. the difference between wake and sleep onset), and efficiency (i.e. sleep duration as a percentage of time in bed) were estimated using a triaxial accelerometer (AX3, Axivity, Newcastle Upon Tyne, UK) worn by participants on the non-dominant wrist for the duration of the study with the exception of during water-based activities, showers and baths). Accelerometers were programmed to measure acceleration at 50 Hz (dynamic range of ±8 *g*). Non-wear periods were defined as either 1) a window of at least 1 hour with less than 13 *mg* for at least 2 out of 3 axes, or 2) where 2 out of 3 axes measured less than 50 *mg* ^28^. Sleep windows were assessed using methods described elsewhere ^39^.

### PREDICT 2 and PREDICT 3 studies.

PREDICT 2 (n=971) (NCT03983733) and PREDICT 3 (n=4562) (NCT04735835) studies took place after the PREDICT 1 study had concluded. The Predict 2 and 3 studies were remote interventions carried out in the United States (US). Ethical approval was granted by the Mass General Brigham Institutional Review Board (PREDICT 2: no. 2018P00207; PREDICT 3: no. 00000971). The informed consent and ethical committee approvals covered all analyses reported here. The studies were run in accordance with the Declaration of Helsinki and good clinical practice; and all participants provided informed written consent. Demographic information, dietary data, cardiometabolic blood biomarkers, and postprandial responses to standardized test meals were collected in PREDICT 2 and 3. Key differences from the PREDICT 1 study include; for body composition measures, height and weight were self-reported for PREDICT 2 and 3. Fasting and postprandial lipaemic responses were assessed using whole blood finger-prick samples collected at home using dried-blood spots and total cholesterol, HDL-C, TG and HbA1c were measured using Quest Diagnostics. CGMs were fitted at home by participants; PREDICT 2 participants were not informed of data availability but some participants became aware that real time CGM glucose readings were available. PREDICT 3 participants were informed that CGM glucose readings were available and could check and self-experiment as they pleased. Unblinded participants were included in analysis as they are representative of CGM use in real-world conditions.

**Figure 1|:**
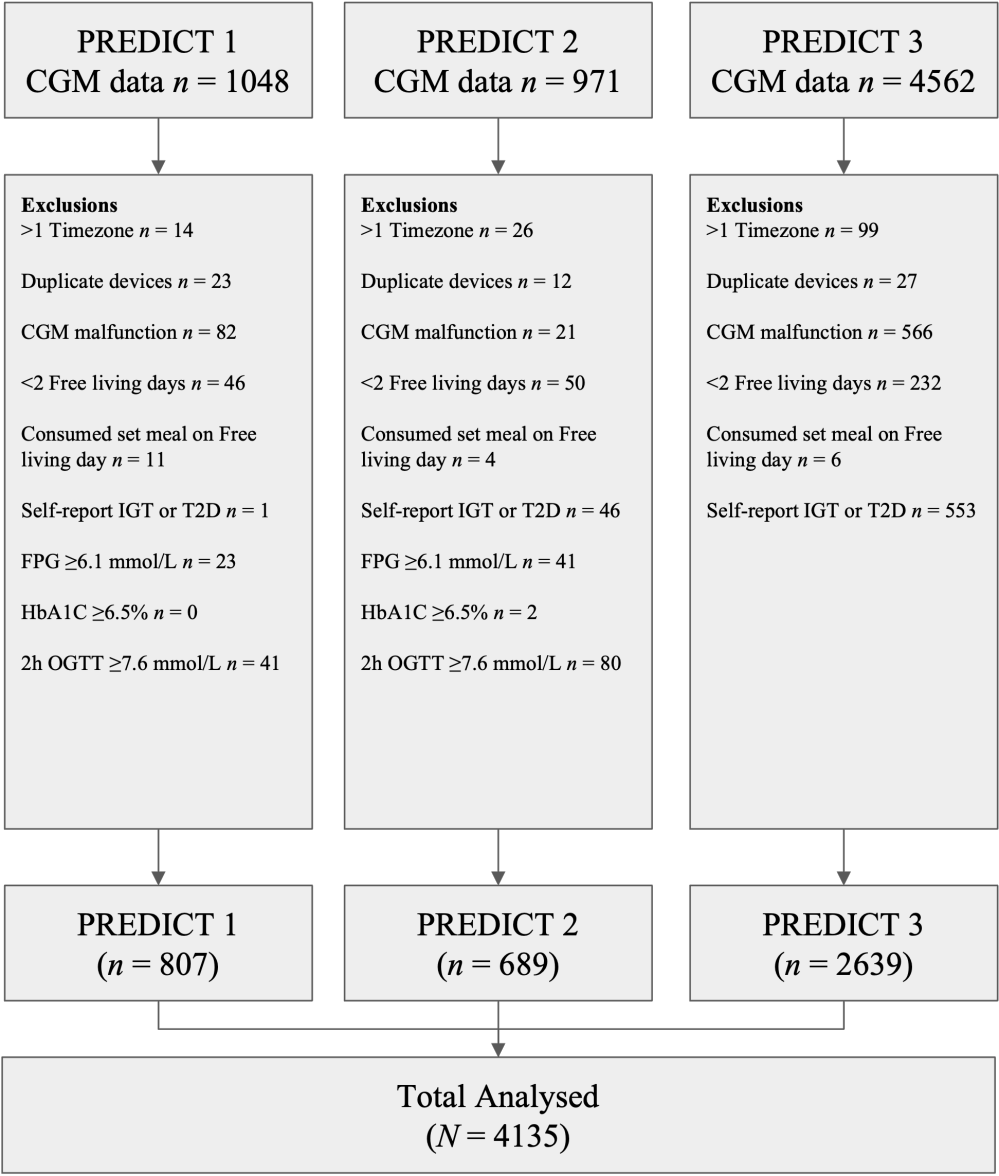
Consort flow diagram illustrating data cleaning process for PREDICT 1, 2 and 3 cohorts. FPG = Fasting Plasma Glucose; IGT = Impaired Glucose Tolerance; OGTT = Oral Glucose Tolerance Test.

PREDICT 1, 2 and 3 cohorts were included in this analysis. The final cohorts used for this analysis, after exclusion, consists of participants without diabetes, IGT (fasting glucose ≥6.1 mmol/L or OGTT ≥7.6 mmol/L) or HbA1C ≥6.5% (48 mmol mol-1), with valid CGM data across ≥2 free-living days (Figure 1) (N=4181; PREDICT 1: n=807; PREDICT 2: n=735; and PREDICT 3: n=2639).

### Statistical analysis.

All statistical analyses were performed using *R* (version 3.4.2, *R* Core Team). Descriptive characteristics, including baseline characteristics and fasting blood biomarkers, are summarised in **Supplementary Table 5**. Differences in participant characteristics and CGM glycaemic metrics (daily glucose, glycaemic variability and TIR) were assessed between cohorts using one-way analysis of variance. Top and bottom quintiles of TIR and glycaemic variability were selected to permit stratification across sex, age, BMI, HbA1c, and lipids (total cholesterol, HDL-C and TG) and differences between quintiles were tested using Mann Whitney U tests. Correlations between dietary intakes (habitual and free-living), dietary habits or lifestyle factors (e.g. activity and sleep) and TIR and glycaemic variability were assessed using correlation analysis while adjusting for age, sex, BMI and diet quality (PDI) using the “*ppcor”* package. The Benjamini-Hochberg correction for multiple comparisons was applied and statistically significant thresholds were based on FDR cut-offs (*q*< 0.05) ^40^. To determine whether the number of days with CGM data influenced glycaemic metrics, we correlated incremental sampling periods of CGM data. We tested correlations between glycaemic variability and TIR_3.9–5.6_ calculated using varying numbers of days in a sub cohort of PREDICT 1 participants with up to 4 free living days (n=175): specifically Spearman’s correlations between glycaemic metrics (glycaemic variability and TIR_3.9–5.6_) calculated using 1 *vs* 4 days, 1 *vs* 3 days, 2 *vs* 2 days, 2 *vs* 3 days, 2 *vs* 4 days and 3 *vs* 4 days were explored (**Supplementary Figure 1**) with good estimates across all days ranging from *r*_s_; 0.65–0.99. Receiver operating characteristic (ROC) curves were constructed and ROC-area under the curve (ROC-AUC) was calculated to assess the discriminatory accuracy of glycaemic metrics to detect ASCVD 10-year risk, Homeostatic Model Assessment for Insulin Resistance (HOMA-IR) and glycoprotein acetylation (GlycA) (70% applied as a cut-point). ROC-AUC ranges from 0.5 to 1.0, with 0.5 indicating no discrimination, and 1.0 indicating perfect discrimination. *P* ≤ 0.05 was considered statistically significant.

## Results

Descriptive characteristics for the PREDICT cohorts are presented in Table 1. Cohorts were predominantly female (PREDICT 1; 75%, 2; 73%, 3; 88%) and HbA1c concentrations were below levels for people living with diabetes (6.5%, 48 mmol/mol) for all cohorts (PREDICT 1; 5.5 ± 0.3% (37 mmol⋅mol-1), PREDICT 2; 5.2 ± 0.3% (33 mmol/mol), PREDICT 3; 5.2 ± 0.5% (33 mmol⋅mol-1)). Mean daily glucose, as measured by CGM, was 4.93 ± 0.42 mmol⋅L-1, 4.99 ± 0.42 mmol⋅L-1 and 5.19 ± 1.11 mmol⋅L-1 in PREDICT 1, 2 and 3 respectively.

### Glycaemic variability and TIR

**Figure 2|:**
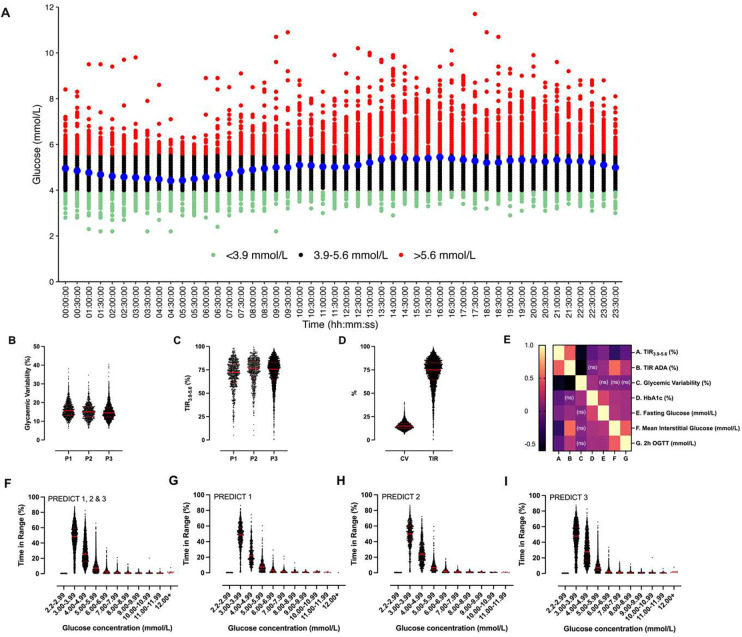
Variation in glucose measured by CGM. A) glucose concentrations in a 24 hour period in the ZOE PREDICT 1, 2 and 3 studies (n=4181). Red points mark glucose values >5.9 mmol/L, black points mark glucose values between 4.0–5.9 mmol/L and green points mark glucose values ≤3.9 mmol/L. B) Distribution of glycaemic variability in the PREDICT 1 (n=807), PREDICT 2 (n=735) and PREDICT 3 cohorts (n=2639). C) Distribution of glucose TIR (3.9–5.6 mmol/L) in the PREDICT 1, 2 and 3 cohorts. D) Distribution of glycaemic variability and TIR in the combined cohorts (PREDICT 1, 2 and 3, n=4181). E) Correlation matrix for CGM-derived and clinical measures of glycaemia. F) Time spent in incremental 1 mmol/L ranges for PREDICT 1, 2 and 3. G) Time spent in incremental 1 mmol/L ranges for PREDICT 1. H) Time spent in incremental 1 mmol/L ranges for PREDICT 2. I) Time spent in incremental 1 mmol/L ranges for PREDICT 3. For figures B, C, D, F, G, H & I the red solid lines denote the median for each violin plot with interquartile range denoted by red dashed lines.

Daily fluctuations in interstitial fluid glucose concentrations under free living conditions are shown in Figure 1A. Median glycaemic variability and TIR_3.9–5.6_ in PREDICT 1 were 15.8% (IQR 13.2–18.7) and 72.4% (IQR 62.0–80.7) respectively (Figure 2B and 2C and Table 1). Similar glycaemic variability and TIR_3.9–5.6_ values were observed in PREDICT 2 (15.0% [IQR 12.5–17.7] and 76.6% [IQR 66.7–83.9]) and PREDICT 3 (14.5% [IQR 12.3–17.2] and 75.7% [IQR 64.6–83.0]). Time spent within and outside ranges are shown in Table 1, with time spent in 1 mmol L^−1^ incremental glucose concentrations shown in Figure 2F–I. There was large inter-individual variation in glycaemic variability (PREDICT 1: 26.2%; PREDICT 2: 26.0% and PREDICT 3: 27.2%). Inter-individual variability in TIR_3.9–5.6_ (PREDICT 1: 22.7%; PREDICT 2: 19.7% and PREDICT 3: 21.4%). was higher than that for TIR_ADA_ (PREDICT 1: 15.3%; PREDICT 2: 15.0% and PREDICT 3: 13.0%).

Spearman’s correlation coefficient between the CGM-derived glycaemic metrics (Figure 2E) was used to elucidate the degree to which different glycaemic metrics were related in participants without diabetes (PREDICT 1 cohort only, n=807). The strongest correlations between two glycaemic metrics were found between TIR_ADA_ and glycaemic variability (*r*_s_ = −0.60, 95% CI: −0.65 – −0.55). Glycaemic variability and TIR_3.9–5.6_, were modestly inversely correlated (*r*_s_ = −0.52, 95% CI: −0.57 – −0.46). The correlation between mean daily glucose concentration and HbA1c levels was weaker (*r*_s_ = 0.15, 95% CI: 0.08 – 0.22). HbA1c was also modestly associated with TIR_3.9–5.6_ (*r*_s_ = −0.15, 95% CI: −0.22 – −0.08) but not TIR_ADA_ (*r*_s_ = −0.04, 95% CI: −0.14 – 0.00).

### Relationship of diet and lifestyle with CGM glycaemic metrics in the PREDICT 1 cohort

To explore the relationship between diet and glycaemic metrics, both habitual (FFQ) and free-living (logged) dietary data were assessed in PREDICT 1 (*n*=807). On free-living days with corresponding CGM data, protein, carbohydrate, and fat intakes (logged diet data derived) contributed on average 15.8 ± 4.9 %, 43.1 ± 10.0 % and 36.3 ± 9.0 % to total energy intake respectively; mean fibre intake was 4.6 ± 12.6 g total fibre per 1,000 kcal. (**Supplementary Table 1**).

**Figure 3|:**
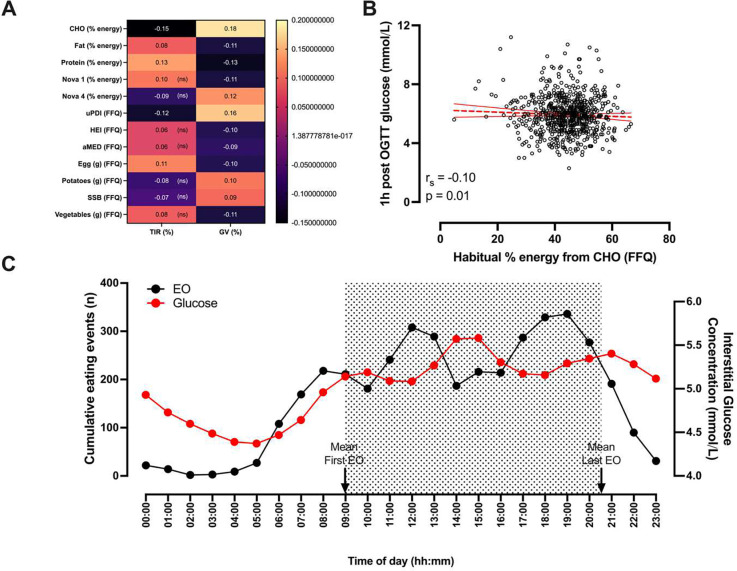
A) Selection of associations between measures of dietary intake and TIR_3.9–5.6_ and glycaemic variability. B) Association (Spearman correlations) between 1h post oral glucose load glucose concentration and habitual carbohydrate intake (% energy intake) as determined by the FFQ C) Fluctuations in eating events and interstitial fluid glucose concentrations across a day. Black dots correspond to the right axis and are mean interstitial fluid glucose concentrations (mmol/L). Red dots correspond to the left axis and are the mean number of eating events (≥50 kcal) in the PREDICT 1 cohort, peaks correspond to main meal times (breakfast, lunch and dinner) throughout the day. The grey shaded area marks the cohorts eating window delineated by the average first and last eating occasion times (defined as any occasion where a food or beverage was consumed that contained ≥50 kcal and was separated in time from the preceding and succeeding EO by 30 minutes ^35,36^).

Higher glycaemic variability was correlated with an unfavourable diet quality index (unhealthy plant based diet index (uPDI), r_s_: 0.16) and higher intakes of carbohydrate (% energy intake (EI ), r_s_: 0.17), ultra-processed foods (NOVA 4, % EI, r_s_: 0.12), potatoes (r_s_: 0.10) and sugar-sweetened beverages (r_s_: 0.09) (all p < 0.05, adjusted for age, sex, BMI, and energy) (Figure 3A). Conversely, a lower glycaemic variability was associated with favourable diet quality indexes (Healthy Eating Index (HEI) [r_s_: −0.10], Mediterranean diet score (aMED) [r_s_: 0.16]), as well as higher intakes of protein (%EI, r_s_: −0.13), fat (%EI, r_s_: −0.11), unprocessed foods (NOVA 1, %EI, r_s_: −0.11), vegetables (r_s_: −0.11) and eggs (r_s_: −0.10, p<0.05 for all). TIR_3.9–5.6_ was more weakly associated with diet, but was also positively associated with intakes of protein (%EI, r_s_: 0.13) and eggs (r_s_: 0.11) and negatively associated with carbohydrate intakes (%EI, r_s_: −0.15) and unhealthy diet quality (uPDI) (r_s_: −0.12, all p<0.05). Of note, there were no significant associations between dietary intake and mean daily interstitial fluid glucose (CGM-derived), and fasting plasma glucose (venous blood sample) was only associated with habitual alcohol intake. HbA1c was positively associated with carbohydrate (%EI) and sugar (%EI) intake whereas interstitial glucose concentration 2h post OGTT was not associated with diet (**Supplementary Table 1**). Higher habitual carbohydrate intake (FFQ derived) was associated with lower OGTT postprandial glucose response (1hr concentration) but was inversely correlated with TIR (Figure 3B).

We also examined timing-related free-living dietary habits including number of eating occasions, cumulative number of main meals and snacks, fasting window (hrs), first and last eating occasion (EO) (clock time), and eating midpoint (midpoint between first and last EO), whilst adjusting for age, sex, BMI and energy intake (kcal) (Figure 3C). Longer overnight fasting windows (average duration across free-living days) were associated with lower glycemic variability (r_s_: −0.10, p = 0.01). The time of the first EO was positively associated with the fasting window (r_s_: 0.68), signifying later breakfast leads to longer fasting duration (**Supplementary Table 2**). To understand whether commencing the eating window earlier versus later independent of fasting time was favourable to glycaemia we adjusted for the fasting window; the time of first EO and last EO were no longer associated with glycaemic variability and TIR_3.9–5.6_ after adjustment, respectively. Next, we examined point-in-time analysis, examining daily glycaemic responses (glycaemic variability and TIR_3.9–5.6_) with preceding day overnight fasting windows. Glycaemic variability was inversely associated with the preceding day fasting window (r_s_:−0.08, p = 0.03). No other associations were evident with the other timing-related free living dietary habit variables.

TIR_3.9–5.6_ was inversely associated with activity energy expenditure (AEE, kcal) (r_s_: −0.09, p = 0.03), but not with sleep duration or efficiency (**Supplementary Table 3**). However, glycaemic variability was not associated with physical activity or sleep metrics. Mean daily interstitial glucose concentrations were positively associated with AEE (rs: 0.13, p < 0.001), and negatively associated with sleep duration (rs: −0.09, p = 0.02). HbA1c was negatively correlated with sleep duration only (r_s_: −0.09, p = 0.02). No other associations were evident between glycaemic metrics, physical activity, and sleep.

### Stratification of baseline characteristics and clinical markers on TIR and glycaemic variability

**Figure 4|:**
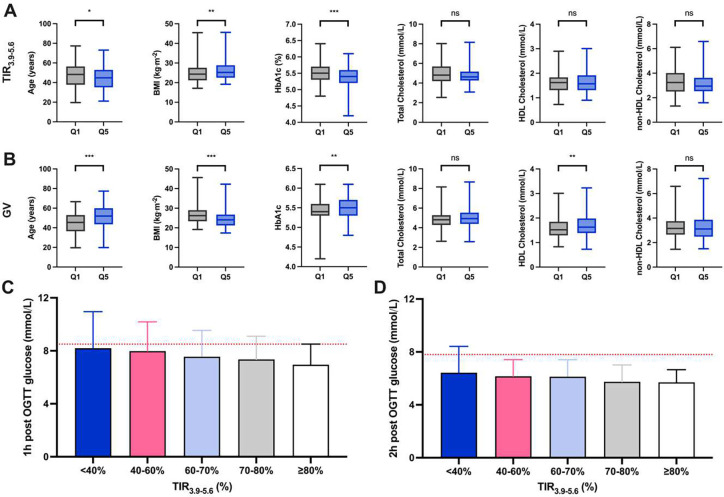
Stratification in the PREDICT 1 cohort (n=807) by sex (males and females), age (years), BMI (kg/m^2^), HbA1c (%), total cholesterol (mmol/L), HDL-C (mmol/L) and TG (mmol/L) by top and bottom quintiles of A) TIR (%), B) glycaemic variability (%). Glucose concentrations post oral glucose tolerance test (OGTT) at C) 1h and D) 2h timepoints across different percentage times in ranges (exploratory targets 3.5–5.6mmol/L). Red dotted lines mark dysglycemia cut-offs, including 7.8 mmol/L (impaired glucose tolerance) at 2h and 8.5 mmol/L at 1h.

Baseline characteristics and clinical chemistry measurements were stratified based on variation in TIR_3.9–5.6_ and glycaemic variability, and the top (Q5) and bottom (Q1) quintiles of both glycaemic metrics were compared (Figure 4).

In the PREDICT 1 cohort, more favourable glycaemic control (i.e. greater TIR_3.9–5.6_ and lower GV) was associated with younger age (median difference TIR_3.9–5.6_: 3 y, GV: 6 y), and lower HbA1c (median difference 0.10%), (median difference TIR_3.9–5.6_: 0.10 %, GV: 0.10 %). More favourable glycaemic variability was also associated with higher BMI (median difference TIR_3.9–5.6_ 0.87 kg/m^2^, GV; 2.15 kg/m^2^), however this relationship did not persist when controlling for energy intake from fat (p > 0.05) (Figure 4A and 4B). Additionally, lower glycaemic variability was associated with lower HDL-C (by 0.11 mmol/L) (Figure 4B). Triglycerides were not different across groups. Similar trends were observed in the PREDICT 2 and 3 cohorts (**Supplementary Figure** 2). A linear trend was also found between TIR_3.9–5.6_ and glycaemic responses to an OGTT (1h and 2h concentrations) (Figure 4C and 4D). Participants (6%) with TIR <40% had a mean 1h post OGTT glucose of 8.20 2.75 mmol/L compared to participants (%) with TIR_3.9–5.6_ ≥80% who had a 1h post OGTT of 6.95 1.56 mmol/L.

### Glycaemic metrics in relation to surrogate health scores

In PREDICT 1, differences in HbA1c, ASCVD 10y risk and HOMA-IR between quintile 1 and quintile 5 of TIR_3.9–5.6_ were apparent that were not observed between the lower and upper quintiles of TIR_ADA,_ or glycaemic variability (**Supplementary Table 5**).

**Figure 5|:**
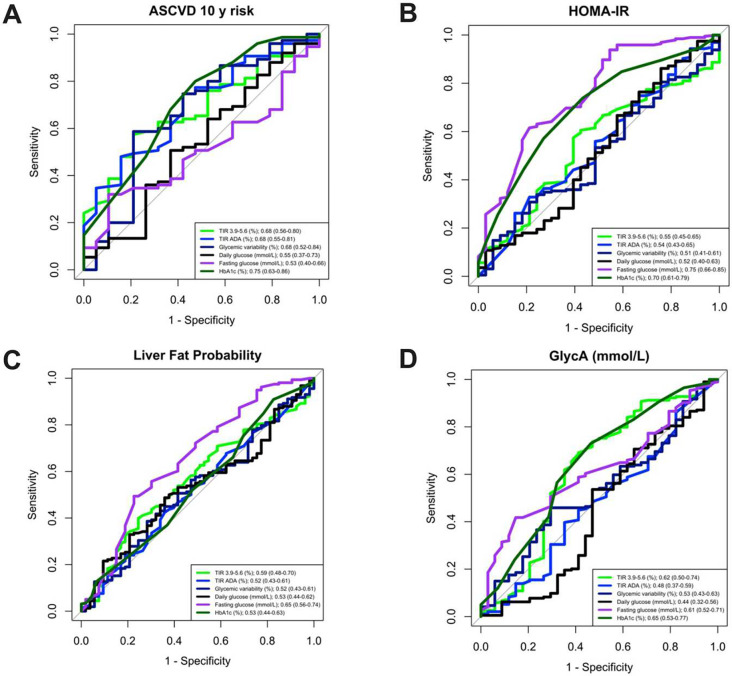
Associations between TIR_3.9–5.6_ (%),_,_ TIR_ADA_ (%), glycaemic variability (mmol/L), fasting glucose (mmol/L) and HbA1c (%) with clinical measures A) ASCVD risk B) HOMA-IR C) Liver Fat Probability and C) Inflammation (GlycA)) in the PREDICT 1 cohort. ROC curves illustrating the utility of glycaemic metrics to distinguish the bottom 70% from the top 30% of the cohort

The ROC-AUC was compared to examine the extent to which glycaemic metrics (TIR_3.9–5.6_ (%),_,_ TIR_ADA_ (%), glycaemic variability (mmol/L), fasting glucose (mmol/L), and HbA1c (%)) could be used to identify individuals with intermediary cardiometabolic disease risk measures, including ASCVD 10-year risk (ASCVD cut-off 5%), insulin resistance (HOMA-IR cut-off 1.9), liver fat probability (cut off 0.23) as well as fasting inflammation (GlycA cut-off 1.4 mmol/L) (cut-offs selected based on clinical guidelines [41, 42]). TIR_3.9–5.6_ was moderately effective at identifying ASCVD risk (ROC-AUC = 0.68 (95% CI 0.56–0.80)), HOMA-IR (ROC-AUC = 0.55 (95% CI 0.44–0.65)), Liver Fat Probability (ROCAUC = 0.59 (95% CI 0.48–0.70)), and inflammation (ROC-AUC = 0.62 (95% CI 0.50–0.74)). In comparison, TIR_ADA_ was also moderately effective at identifying ASCVD risk (ROC-AUC = 0.69 (95% CI 0.57–0.82)) and HOMA-IR risk (ROC-AUC = 0.54 (95% CI 0.43–0.65)), but was less effective than TIR_3.9–5.6_ for identification of Liver Fat Probability (ROC-AUC = 0.52 (95% CI 0.43–0.61)), and inflammation (ROC-AUC = 0.48 (95% CI 0.37–0.59)). Glycaemic variability was less effective in comparison to TIR_3.9–5.6_ for identifying HOMA-IR (ROC-AUC = 0.51 (95% CI 0.41–0.61)), Liver Fat Probability (ROC-AUC = 0.52 (95% CI 0.43–0.61)) and inflammation (ROC-AUC = 0.53 (95% CI 0.43–0.63)), but was moderately effective for identifying ASCVD (ROC-AUC = 0.68 (95% CI 0.520.84)) (Figure 5). HbA1C was the most effective at identifying risk for ASCVD (ROC-AUC = 0.71 (95% CI 0.57–0.84)), Insulin Resistance (HOMA-IR; ROC-AUC = 0.70 (95% CI 0.61–0.79)) and inflammation (ROC-AUC = 0.65 (0.53–0.77)), but not Liver Fat Probability (ROC-AUC = 0.53 (95% CI 0.44–0.63)). Fasting glucose was better than TIR_3.9–5.6_ for HOMA-IR (ROC-AUC = 0.75 (0.66–0.85), and was also the strongest predictor of Liver Fat Probability (ROC-AUC = 0.65 (95% CI 0.56–0.74)).

## Discussion

These analyses investigated continuous interstitial fluid glucose profiles in participants without diabetes or impaired glucose tolerance using CGMs. Under free living conditions, median glycaemic variability (coefficient variation) was ~15%, and individuals spent ~96% of their time with glucose values between 3.9 and 7.8 mmol/L, compared to ~75% of time within an exploratory TIR target for people without diabetes (3.9 – 5.6 mmol/L). This exploratory TIR target was sensitive to changes in HOMA-IR and is discriminatory of ASCVD risk despite normal HbA1c concentrations. Equally, this proposed range was also associated with diet indices relating to both quantity and quality of foods consumed, as well as patterns of feeding and fasting.

### A more stringent TIR target provides better discriminatory potential than established ranges to detect changes in cardiometabolic health in individuals without diabetes.

In the present study we found participants across three cohorts spent ~ 95% of their time within the established secondary ADA TIR target (3.9–7.6 mmol/L) ^2^. This is in line with a series of previous studies showing that, on average, metabolically healthy individuals spend 93–99% of their time within this range ^10–19^. However, both fasting and non-fasting concentrations of glucose >5.6 mmol/L are associated with increased risk of diabetes and associated comorbidities highlighting the need to revisit current glycaemic targets for healthy individuals ^20–22^. One previous study reports ~20% of time is spent above 5.6 mmol/L, while ~80% was spent between 3.3 to 5.6 mmol/L ^10^; comparatively TIR_3.9–5.6_ was ~75% in the current study. The findings of the current study are in agreement with those of Keshet *et al* ^19^ in reporting associations between glycaemic metrics and markers of CVD risk. Comparison of upper and lower quintiles of TIR_3.9–5.6_ revealed differences in HbA1c, ASCVD 10y risk and HOMA-IR, that were otherwise not observed using TIR_ADA_. This hints at the potential superior discriminatory potential of utilising a stringent TIR in a population without diabetes or IGT; in the case of HOMA-IR, this might suggest CGM *could* be a useful tool for identifying insulin sensitivity without the need to measure insulin, however further work is required to confirm this. The current study further builds on the work of Keshet *et al* ^19^ through assessment of responses to standardised OGTT. A longer time spent in stringent ranges was associated with a linear decrease in glucose concentrations away from unfavourable diagnostic ranges for both a 2h and 1h OGTT glucose concentrations. For the latter, glucose ≥8.5 mmol/L is suggested to be predictive of diabetes progression and increased risk for microvascular disease and mortality ^41^, whereas for the former, a 2 h postprandial glucose above fasting is associated with coronary heart disease and ischemic stroke in individuals with normo-glycaemia ^8^. Collectively these data suggest that CGMs may be useful in early identification of changes in intermediary cardiometabolic disease risk measures, such as ASCVD and HOMA-IR, which typically precede changes in most other risk measures ^42^. In turn this may help to prevent development of impaired glucose tolerance as well as progression to prediabetes and diabetes ^4,41^.

### Lower carbohydrate intake is associated with improved daily glycaemic variability, but chronic low carbohydrate intake may reduce measured glucose tolerance.

Under controlled conditions, postprandial glucose responses are dependent on both physiological factors (insulin secretion, insulin sensitivity and glucose effectiveness) as well as dietary intake (carbohydrate content and glycaemic index of foods) and contextual factors (sleep, physical activity, stress, time of day) ^43^. In free living settings, large day-to-day variability exists in dietary intake as individuals consume different food combinations in varying amounts at random times and meals are consumed in different proximity to other meals, overnight fasting, physical activity and sleep ^23,25^. To understand some of the complexity in dietary data we examined both habitual and free-living dietary intakes in relation to glycaemic variability and TIR_3.9–5.6_. Protein intake was positively associated with TIR_3.9–5.6_ and inversely associated with glycaemic variability, agreeing with previous evidence showing beneficial effects of protein on glycaemic outcomes ^19,24,44^. Logged carbohydrate intake was negatively associated with TIR_3.9–5.6_ and positively associated with glucose variability suggesting that the greater carbohydrate intake worsens daily glycaemic responses, consistent with Keshet *et al* ^19^. Habitual carbohydrate intake, on the other hand, was negatively correlated with 1-h postprandial glucose during an OGTT, further highlighting the relationship between habitual carbohydrate intake and improved measured glucose tolerance ^45–50^. Taken together, these data demonstrate the uncoupling of dietary factors between OGTT-derived versus CGMs-derived glycaemic metrics suggesting that acute exchange of carbohydrate with protein is associated with lower glycaemic variability, but chronic adaptation to such diets may not reflect acute responses.

Food preparation and processing techniques have also been shown to influence postprandial glycaemic responses, due to modifications of starch structures and the food matrix ^26,51^. However, to the best of our knowledge this is the first study to demonstrate an association between intake of NOVA classified “ultra-processed” foods and glycaemic variability. More specifically, intake of NOVA category 1 foods was inversely associated with glycaemic variability, intake of NOVA category 4 foods was positively associated with glycaemic variability. Furthermore, habitual intake of sugar-sweetened beverages positively correlated with glycaemic variability, yet there was no clear evidence of a correlation between habitual sugar intake per se and glycaemic variability. This supports the hypothesis that the types of foods ingested mediate the glycaemic responses to sugars, however further work is required to establish the exact mechanisms of this effect. These data add to the growing body of literature suggesting that foods are not the simple sum of their nutrients and quality, processing and formulation are essential considerations when characterizing foods and considering their effects on health.

### Cycles of feeding and fasting influence daily glycaemic variability.

Longer nocturnal fasting duration was associated with lower glucose coefficient of variation (Supplementary Table 3). This is consistent with the long/late eating windows and may suggest a shorter eating window is associated with favourable daily glycaemic variability ^52,53^. Several time restricted eating (TRE) studies in adults have demonstrated improvement in glycaemic metrics such as mean daily glucose, fasting glucose, and postprandial glycaemic responses compared to those eating over prolonged time windows ^54–57^; however, others have found no TRE treatment effect on glycaemic metrics ^58–61^, or worsened glycaemic variability ^62^. This may be related to the time of day in which eating and/or periods occur ^23,62^; none-the-less further investigation is warranted to determine the exact mechanism of these inconsistencies. Future research incorporating additional elements of the diet such as fasting/feeding windows into dietary recommendations may provide additional avenues through which glycaemic health can be maintained.

Finally, we explored the relationship between physical activity and sleep with TIR and glycaemic variability. Greater activity energy expenditure (AEE; kcal) was associated with lower TIR. This was surprising given the established effects of physical activity on improving TIR and GV in individuals with T2D ^63,64^. However there are several potential explanations for a lack of effect in the current study. Firstly, a ceiling effect of physical activity may exist in non-diabetic individuals whereby little additional improvement is seen with increasing activity energy expenditure on top of the improvement in glycaemic regulation seen from reducing sedentary time ^64,65^. Secondly, it is possible that activity levels on free-living days were not reflective of those typically seen in the study cohort, due to the intensive testing protocol in PREDICT 1 ^28^. Nevertheless, mean daily interstitial glucose concentration was positively associated with AEE, however the causality of this relationship is not clear and future research should establish whether high levels of physical activity drive higher glucose levels or vice versa.

Our previous work revealed the effects of sleep duration on the acute glycaemic responses (2h iAUC) to set meals measured consumed under standardised conditions using CGMs ^25^. The current study therefore extends on this by exploring the influence of both sleep duration and sleep efficiency on whole day glycaemic metrics under free-living conditions. Neither sleep duration nor efficiency were related to TIR and GV but mean interstitial glucose concentration was inversely related to both sleep metrics. Taken together this might suggest that whilst lack of sleep perturbs the underlying physiology that regulates glycaemic control ^66,67^, the influence of behavioural factors may attenuate dysregulated blood glucose control in a free living context ^68^.

Limitations of this analysis include: 1) cross-sectional data preventing identification of causal relationships; 2) low number of free living days of CGM data; 3); no data on female menstrual cycle status, which may influence insulin sensitivity and therefore indirectly TIR, 4) some participants were unblinded to CGM results which potentially biased CV and TIR metrics from PREDICT 2 and 3 cohorts. The data presented examines markers of cardiometabolic health, postprandial metabolism, metabolic syndrome factors, and diet across the deeply phenotyped PREDICT 1 cohort.

In summary, individuals with normo-glycaemia spend ~75% of their time within a stringent range of interstitial glucose concentrations (3.9–5.6 mmol/L) compared to ~96% of time within the established ADA normative glucose range (3.9–7.8 mmol/L). This stringent time in range target provides sensitivity to detect changes in HOMA-IR, ASCVD 10 y risk and HbA1c that ADA targets are otherwise unable to. Additional novel associations among TIR, glycaemic variability and aspects of diet and lifestyle (e.g. NOVA 4 categorisation, protein intake, nocturnal fasting duration) were also observed which highlight potential dietary strategy targets to improve glycaemic metrics derived from continuous glucose monitors.

## Figures and Tables

**Table 1 -:** Descriptives of the ZOE PREDICT cohorts

	PREDICT 1*			PREDICT 2			PREDICT 3			p-value		
	n	Mean	SD	n	Mean	SD	n	Mean	SD	P1 vs P2	P1 vs P3	P2 vs P3

Sex (% female)	807	75	-	682	73	-	2639	88	-	0.43^#^	<0.001^#^	<0.001^#^
Ethnicity (% white)	807	79	-	689	69	-	2639	93	-	<0.001^#^	<0.001^#^	<0.001^#^
Age (years)	807	47	13	682	43	12	2636	48	11	0.13	<0.001	<0.001
Height (m)	807	1.68	0.11	682	1.70	0.13	2639	1.67	0.08	<0.01	<0.05	0.50
Weight (kg)	807	72.0	15.0	681	74.6	17.1	2639	78.7	17.9	<0.01	<0.01	0.50
BMI (kg/m^2^)	806	25.4	4.8	681	25.7	5.5	2639	28.2	5.9	0.30	<0.001	<0.001
***Fasting bloods***												
Glucose (mmol/L)	807	4.93	0.42	674	4.98	0.42	2012	5.19	1.11	<0.001	<0.001	0.67
HbAlc (%)	806	5.5	0.3	674	5.18	0.25	2012	5.2	0.4	<0.001	<0.001	0.56
Cholesterol (mmol/L)	807	4.93	0.96	686	4.79	0.92	2012	5.29	1.01	0.89	<0.001	<0.001
HDL-C (mmol/L)	807	1.65	0.42	686	1.60	0.37	2012	1.71	0.49	0.61	<0.01	<0.001
TG (mmol/L)	807	1.05	0.5	686	0.96	0.47	2012	1.92	1.18	0.11	<0.001	<0.001
***CGM measures***		Median	IQR		Median	IQR		Median	IQR			
						
Mean daily glucose (mmol/L)	807	4.99	4.64–5.01	689	4.97	4.67–5.27	2639	5.03	4.70–5.37	0.98	<0.001	<0.001
Glycaemic variability (%)	807	15.8	13.2–18.7	689	15.1	12.6–17.9	2639	14.5	12.3–17.2	<0.05	<0.001	0.18
TIR3.9–5.6 (%, 3.9–5.6 mmol/L)	807	72.4	62.0–80.7	689	76.6	66.7–83.9	2639	75.7	64.6–83.0	0.02	0.95	0.01
Time below range (% <3.9 mmol/L)	807	1.6	0.0–10.4	689	2.1	0.0–9.4	2639	1.7	0.0–7.6	0.22	<0.001	0.13
Time above range (% >5.6 mmol/L)	807	18.2	10.4–28.7	689	15.1	8.9–24.5	2639	17.4	9.7–28.0	0.93	0.01	<0.01
TIR ADA (%, 3.9–7.8 mmol/L)	807	95.3	87.0–98.4	689	96.1	90.1–98.7	2639	96.2	90.3–98.6	0.32	<0.01	0.25
Time above range (% >7.8 mmol/L)	807	0.3	0.0–2.1	689	0.0	0.0–1.6	2639	0.3	0.0–1.7	0.29	0.03	0.75

* PREDICT 1 and cardio combined

# Chi-Squared

## Data Availability

The data used for analysis in this study are held by the Department of Twin Research at King’s College London and access can be requested from https://twinsuk.ac. uk/resources-for-researchers/access-our-data/ to allow for anonymisation and compliance with GDPR standards.
